# Crafting the future of chiropractic research in the UK: designing sustainable practice-based research networks through qualitative exploration and logic model

**DOI:** 10.1186/s12998-026-00630-6

**Published:** 2026-02-19

**Authors:** Michelle M. Holmes, Amy Miller, Dave Newell

**Affiliations:** 1 Centre for Workforce and Systems Innovation, Health Sciences University, Bournemouth, UK; 2 AECC School of Chiropractic, Health Sciences University, Bournemouth, UK

**Keywords:** Practice-based research network, Health services research, Community networks, Workforce

## Abstract

**Background:**

Compared to other health professions, the chiropractic profession lacks a substantive research culture and research capacity, including limited involvement of chiropractors and low recruitment in practice-based research. One recommendation is the establishment of Practice-Based Research Networks (PBRN). This research aimed to explore the feasibility and models of PBRNs informing recommendations for the UK and the wider chiropractic profession.

**Methods:**

Qualitative interviews with key stakeholders explored potential barriers and opportunities of PBRNs in the UK chiropractic setting. Participants included UK chiropractors, UK academics, representatives from organisations in the UK, and international researchers who had developed or utilised PBRNs. Qualitative findings were analysed using thematic analysis and synthesised with findings of literature on PBRN development, to formalise a model for PBRN development and delivery.

**Results:**

Thirty stakeholders participated in an interview. Five themes were developed from the synthesis of the qualitative themes and literature. This included: (1) Purpose of PBRNs within chiropractic, (2) Models and organisational structure of PBRNs, (3) Financial support for PBRN sustainability, (4) Enhancing chiropractor engagement, and (5) PBRN sub-study considerations. Participants highlighted the benefits of PBRNs to the profession and identified a series of recommendations around PBRN infrastructure and activities, which were developed into a logic model to support future PBRN development and delivery.

**Conclusions:**

This study demonstrates the potential benefits of PBRNs in chiropractic, in the UK and internationally. Although several challenges to their development were identified, opportunities and solutions were offered to facilitate a PBRN in the chiropractic context. This research underpins recommendations for the future development, launch and sustainability of PBRNs within the profession in the UK and beyond.

**Supplementary Information:**

The online version contains supplementary material available at 10.1186/s12998-026-00630-6.

## Introduction

The chiropractic profession lacks a robust research culture and routine clinical data collection in comparison with other healthcare professions, who have embraced evidence-based approaches to practice with sustainable research infrastructure to support research capacity, productivity and impact [1]. There remains a lack of research capacity across the profession, despite calls for more research and previous work developing research priorities for the profession [2–4]. In part, this is due to the majority of chiropractors working in private practice [5]; operating outside mainstream healthcare systems limits access to research funding and training opportunities, with professions operating outside of traditional health services often facing funding challenges [6]. With limited incentives for research participation, there are challenges involving chiropractors in research or securing sufficient patient numbers in practice-based data collection. Additionally, some chiropractors perceive research as detached from real-world practice, designed by academics who overlook clinical realities [7].

This perceived disconnect reduces engagement with research initiatives. Lastly, there is considerable heterogeneity of effort in the UK research environment, partly due to limited knowledge of ongoing research efforts. Ideological differences among chiropractors, and the multiple associations, further exacerbate this, with conflicting views on the profession’s identity and priorities [1]. These long-standing divisions have hindered unified research efforts, consensus on research priorities, and support or funding for collaborative initiatives. Consequently, the profession continues to face challenges in developing a cohesive research framework.

Research capacity is also limited in the UK, both in terms of funding and availability of research-trained chiropractors. Specific funding for chiropractic research is dependent on the UK chiropractic profession, through levies, charitable giving, or ring fencing a proportion of membership fees. In academic institutions offering chiropractic training, resources to support academic staff to acquire high-level research experience is limited [4], which is markedly different from research intensive universities, with a success in highly competitive funding, thriving research culture and impactful research groups. There have been several activities to increase opportunities for chiropractic research, particularly focused on recent graduates and early-career researchers, including the UK Programme for Early Researchers in Chiropractic [8] and the international Chiropractic Academy for Research Leadership programme [9, 10]. Whilst initiatives such as these provide opportunities to develop impactful networks and research outputs, these initiatives are dependent on funding, and as such are limited in the number of individuals who can access the programmes. Reflecting the chiropractic profession internationally, the UK chiropractic research landscape is characterised by a small proportion of clinical and academic researchers [10], who underpin much of the UK chiropractic research productivity and impact.

` One recommendation to increase research capacity within the profession is to establish a Practice-Based Research Network (PBRN) [11]. PBRNs are not new to the chiropractic profession, with previous work providing guidance on conducting practice-based research projects [12–14]. PBRNs serve multiple functions, firstly PBRNs aim to engage clinicians, practices, and researchers to work together on research activity, using data collected within clinical practice to create an evidence-informed culture within primary care [15–17]. A second vital function of PBRNs is to provide real-world clinical data, reflecting health service delivery and primary care practice [18], which is particularly valuable in complex interventions such as chiropractic. The research from PBRNs is designed to answer real-world questions with practitioners driving the research agenda [17, 19, 20]. With the research reflective of clinical practice [17, 21, 22], PBRNs can facilitate evidence-based practice [6, 13], whilst providing a mechanism for translation of research findings into practice [20, 23]. The development of a PBRN also allows professions to build a research culture and develop research capacity [6, 13, 21]. This includes development of a research infrastructure to increase research capacity, collaboration, and outputs [24].

There are several existing PBRNs within chiropractic and other musculoskeletal professions. One successful PBRN is the Australian Chiropractic Research Network (ACORN). Research opportunities within this PBRN have explored a range of areas including, but not limited to, basic science, clinical outcomes for a variety of special populations, public health and health services research [24, 25]. Another is the Practitioner Research and Collaborative Initiative (PRACI), open to 15 complementary medicine professions in Australia, including musculoskeletal practitioners [17]. ACORN and PRACI have provided a blueprint for the development of other PBRNs such as the Swiss Chiropractic PBRN [26].

Whilst literature suggests that PBRNs are beneficial structure to improve research capacity, there is limited comprehensive guidance on developing and sustaining PBRNs. The aim of this project was to explore the feasibility and models of PBRNs informing recommendations for the UK.

## Methods

### Design

This study took a qualitative exploratory approach, utilising semi-structured interviews to obtain an in-depth understanding of stakeholders’ views of the development and delivery of PBRNs within the chiropractic profession. Qualitative themes were reviewed against literature to form a model of PBRN development and delivery. Ethical approval for qualitative interviews was approved by Health Sciences University ethics committee (Ethics number: SOC-0921-001).

### Participants

To provide a comprehensive picture of PBRNs in chiropractic, it was important to sample stakeholders from across the chiropractic profession. This included UK academics (both researchers and educators), UK chiropractic professional organisations, UK based chiropractors, and individuals with experience in chiropractic PBRNs internationally. Purposive sampling was used to invite UK academics and international PBRN researchers to the study; 33 people were invited to participate in qualitative interviews, including 16 stakeholders from across the chiropractic profession, and 17 researchers with experience and expertise in PBRNs internationally. UK chiropractors and representatives from chiropractic professional organisations were recruited using convenience sampling, via researcher networks and social media recruitment. Recruitment, data collection and data analysis were conducted simultaneously, continually reviewing for data adequacy.

### Data collection

Online semi-structured individual interviews were conducted with participants to understand their views on PBRNs and the process of developing and delivering a chiropractic PBRN in the UK. Interviews were undertaken by two post-doctoral academics based in the UK, who have prior experience with qualitative interviews (AM, MH). Due to the small field of chiropractic research, the interviewers were known to many of the participants. A topic guide for the interviews was developed and reviewed by all three authors (see Table 1). In line with a semi-structured, reflexive approach, interview questions were flexible to encourage a discussion-like feel to the interview. Interview questions for the UK chiropractic profession (chiropractors, academics, and organisation representatives) focused on participant understanding of PBRNs, recruitment of chiropractors, and infrastructure for PBRNs. Interview questions for those involved in PBRNs internationally focused on involvement in PBRNs, factors for successful PBRNs, and recruitment of chiropractors. The interviews had an average duration of 37 min. Interviews were recorded with permission from participants and then transcribed verbatim and inputted into the computer-assisted qualitative software NVivo [27].

**Table 1:** Interview topic guide

Interview questions with the chiropractic profession	Interview questions for PBRN researchers
Are you currently or have you ever been involved in research? What is your understanding of PBRNs? What are your thoughts on how a PBRN might work? When building a PBRN, we would need to recruit chiropractors and their practice, how do you think we could recruit them? What type of support would you and your practice need to make participation in research feasible?	Can you take me through your involvement in PBRNs? What are your thoughts about PBRNs in chiropractic? When building a PBRN, you need to recruit clinicians and their practice, what did you think about the recruitment process? What support do you think is needed for PBRNs? What do you think are important things to consider before setting up a PBRN?

### Data analysis and model development

The analysis was conducted following published guidance on thematic analysis, providing a systematic and transparent process of conducting qualitative data analysis [28–30]. Stage 1 involved familiarisation with the data, listening to the audio-recordings, transcription, and re-reading the transcripts, with initial notes made at this stage. Stage 2, initial inductive coding, was conducted by one author (MH), describing the data and interpreting the data content. This was conducted at each stakeholder level (UK academics, chiropractors, PBRN experts, and chiropractic organisations in the UK). After initial coding, 244 codes were developed. After all interviews were coded, codes were examined to identify similarity and differences (Stage 3). Codes were refined during Stage 4, including collapsing codes that were similar constructs by two authors (MH, AM). In this step, themes were generated across the four stakeholder groups, based on broad topics in which codes were clustered.

Stages 5 (refining, defining and naming themes) and 6 (writing up) of Braun and Clarke [28–30] were completed in the context of a broader synthesis combining the qualitative findings and existing literature; the synthesis aimed to formalise a model for PBRN development and delivery, considering the context, mechanisms, and outcomes [31]. During the interview and analysis process, a literature search was concurrently undertaken on PubMed, CINAHL, Cochrane Library, and AMED using keywords and MeSH terms on derivatives of PBRNs. Commentaries, reviews and editorials published in English were eligible for inclusion [32, 33]. Papers were included if they focused on PBRNs development generally, or within primary care or musculoskeletal healthcare. Study titles and abstracts were screened to assess eligibility, followed by screening of full texts by two reviewers (MH and AM). The process is documented in supplementary material [1]. Data extraction from literature was undertaken through note-taking, considering common patterns, and were mapped against the the findings of the qualitative interviews [32, 33].

Potential themes across the literature were reviewed against the qualitative dataset to form a logic model of PBRN development and delivery within chiropractic, including the population, inputs and activities of the PBRN, and the outputs and outcomes. This was based on the logic model for the South Texas Ambulatory Research Network (STARNet) [34]. In addition, qualitative findings on chiropractors’ involvement in research [35] and use of data collection tools [36] were integrated to further refine the model. Several iterations of the model were developed, to ensure that no elements were missing. Textual descriptions summarising the literature are provided for each element of the theory with supporting empirical evidence. Examples and verbatim quotes are presented to best illustrate the dataset and describe the findings. The study has been written in line with the RAMESES guidance for reporting realist syntheses [37] and the COnsolidated criteria for REporting Qualitative studies (COREQ) checklist [38].

## Results

### Overview and participants

Of the 33 people purposively sampled, based on their position in the UK chiropractic academics and organisation representatives or international PBRN experience, 25 agreed to be interviewed, including 14 chiropractic stakeholders and 11 researchers. A further convenience sample of five UK chiropractors participated in interviews, following their response to online recruitment. The thematic analysis resulted in findings being organised into nine themes. Within the synthesis, the qualitative findings were mapped against the literature and organised into the following final themes: (1) Purpose of PBRNs within chiropractic, (2) Models and organisational structure of PBRNs, (3) Financial support for PBRN sustainability, (4) Enhancing chiropractor engagement, and (5) PBRN sub-study considerations. The following sections describe each of the findings, noting stakeholder views, with example quotations from participants.

### Purpose of PBRNs within chiropractic

Participants from the qualitative interviews identified a lack of research culture and apathy for research within the chiropractic profession, noting limited involvement from chiropractors in research due to individual knowledge and *“a lack of awareness in terms of what to do to be research active in a clinical setting.”* (P25—academic). This understanding of research was felt to be widespread among the profession, impacting on research capacity: *“I think it's very poorly understood and undervalued generally across the profession.”* (P30—organisation).

Participants compared the chiropractic profession to other healthcare professions: *“There's some still some issues around, you know, identity and reputation etcetera and I think there's a feeling that there's a lack of research”* (P12—academic). Participants highlighted the need for the profession to grow research capacity and provide more evidence to support chiropractic: *“I just worry about the profession and its future if there is limited or a lack of coming together, researching and the profession evolving”* (P17—organisation).

A PBRN was seen as a positive way to build research culture as *“a way of getting the profession engaged”* (P12—academic). A PBRN was defined as a collaboration between clinicians and academics conduct research, consisting of *“a group of clinicians who are interested in research”* (P6—academic) who would like the opportunity to participate in research. PBRNs were thought to ease data collection and provide opportunities for pragmatic research, enhancing the quality and volume of data. The PBRN connects “*researchers with appropriate clinicians for project development and data collection.”* (P26—academic) with data directly from clinical practice highlighting “*from what's happening in the real world”* (P5—chiropractor).

Participants highlighted benefits of PBRNs to move the profession forward “*by collecting certain data it may be useful for wider stakeholders external to the profession that needs certain information in order to trust the profession more”* (P3—chiropractor). However, it was felt that for chiropractors the *“ultimate benefits are seeing some research that is helpful for their practice.”* (P30—organisation), with PBRNs providing an opportunity for more data and evidence focusing on patient care, with one academic stating that the *“main mission has to be geared towards public benefit”* (P26—academic).

### Models and organisational structure of PBRNs

Interviews with PBRN researchers highlighted two different models of PBRNS: a registry model and a sub-study model. The registry model is focused on coordinated regular collection of patient data using a patient record system. The sub-study model design utilises a practitioner PBRN database, with the initial data collection focused on practitioner-reported data.

All stakeholders identified a difference between the two models, acknowledging that although the registry model would provide a larger volume of data, stakeholders felt that this was not feasible. One researcher involved with PBRN development internationally reflected that chiropractors *“are all wedded, emotionally almost, to their favourite clinical practice software”* (P21—researcher). It was noted that to make a registry model operational and for standard data collection processes to be implemented “*you're going to have to bring in some sort of system that doesn't already exist in order to collect data.”* (P9—researcher). Based on the recommendations of PBRN researchers and the challenges raised by chiropractors and chiropractic stakeholders in the UK, the sub-study model was seen as the most appropriate model for current clinical practice.

Participants noted that a PBRN would need organisation and management, requiring “*capacity to operate it, you know, to operate the system, to manage the system. So it's not just setting it up, it's the ongoing work with it, so you know, issues about ownership and licensing updating the system, the administration of it*” (P30—organisation). Many academics agreed that a UK educational institution should take a strategic role in setting up the infrastructure and project management of a PBRN. One academic stated “*Devolving it from the educational institutions, I think would almost be impossible”* (P16—academic).

The additional challenge of the political nature of chiropractic was noted by organisations*.* It was felt that it was important to get leaders of organisations on board to encourage members to get involved. One academic identified that the message should be collaboration across all associations, organisations, and clinicians, recommending *“a big launch at the right time rather than this sort of little trickle effect coming from different people with different messaging. I think it's getting that absolutely clear. Big launch, everyone supporting it”* (P22—academic). Participants identified that organisations could help their members understand the PBRN, what it is, and why it is important. *“Not everyone's obviously a member of a professional association. But if the messages were coming from everybody. Then I think that would be much, much stronger”* (P17—organisation).

### Financial support for PBRN sustainability

Throughout the interviews it was highlighted that a PBRN *“requires an infrastructure”* (P11—researcher). Along with this, researchers involved in PBRN’s internationally stressed the importance of funding, and the funding required for PBRN development, infrastructure and ongoing support. A lack of funding was identified within the literature as a key challenge to supporting PBRN infrastructure and activities: *“If you can’t get funding, then I just don’t think you should do it”* (P10—researcher). International start-up costs varied, although this was noted in the success and long-term sustainability of the project. Researchers noted that these costs varied from £8000 to £200,000 in start-up costs. The start-up costs often related to travel, marketing materials, and attendance at events to market the network. Ongoing infrastructure costs included items such as a director, administrator, marketing materials, travel and meetings.

The ongoing financial support was noted as challenging if dependent on grant funding: *“how do you sustainably maintain this thing? Cos, you know, and we struggle in the research world. I suspect you guys do too, where you can get a great injection of funds to build it. But how do you sustain it?*” (P11—researcher). Organisational representatives in the UK said they might be able to financially support the project as they funded research previously *“the other thing that we do is we sponsor studies. So either a researcher or a university or something come to us with a proposal and we fund it.”* (P29).

The funding for PBRNs also included administration costs. Primary care research and research conducted within PBRNs have several administrative requirements, with the suggestion that some administration should sit centrally *“you wouldn't want to be having to recruit that administrator each and every time. So they've got to be employed by the PBRN*” (P27—organisation). A researcher with previous involvement in PBRNs identified the need for a “*designated central admin person that could keep people engaged and distribute things and set up meetings, that would probably be helpful. Umm, so setting that infrastructure up from the beginning. Rather than having researchers do a lot of that stuff”* (P23—researcher).

### Enhancing chiropractor engagement

Practitioners joining a sub-study model PBRN means consenting to be on a database of practitioners open to receiving invitations for future research projects. A PBRN researcher noted the value in this approach as *“A ready-made army of willing practitioners. And it's a phenomenal resource for any research team to be able to get their hands on”* (P21—researcher). Chiropractors could also be advocates for the PBRN, involved in the recruitment of practitioners and feedback by practitioners to the network: *“A champion basically that just says, come on, you know this, you told me you'd like to participate. This is your chance. And let's do this.” (*P13—researcher).

Common barriers for practitioners to engage with PBRNs include lack of time and competing priorities, low interest in the research topic, and limited research skills. One academic noted that *“there isn't buy in from the profession in that the majority chiropractors don't see that research is part of their job.”* (P25—academic). From their previous experience in engaging chiropractors in research, representatives from chiropractic organisations commented on chiropractors not seeing the benefit of research *“unless it's interesting and inspiring to them and they feel like it's gonna take the profession forward, I just know they won't participate” (P29—organisation).*

Existing PBRNs within chiropractic have used various strategies to engage with the profession. For small PBRNs this involved snowball recruitment *“We just started, yeah, contacting clinicians in the area. Some we knew, some we didn't, and just garnered interest”* (P9—researcher). However, others took a more structured approach to recruitment. Important messages were noted surrounding improving practice: *“enabling clinicians to see the benefit to chiropractic and maybe some benefit to themselves and their patients as well.”* (P30—organisation)”, *“I think there is there is a sense of and instilling into people, giving back to the profession”* (P25—academic). A prior PBRN reflected on the impact this had on recruitment *“the one thing that brought people back to the table all the time was just to focus on the only reason we're all there and that’s patient-centred care”* (P13—researcher).

Several ideas to increase involvement were identified. This included recognising PBRN involvement as continuing professional development, solving two problems *“lot of people do struggle to get their hours*” (P5—chiropractor). Other recommendations included hosting seminars, academic talks, and guest speakers through the PBRN. This also enabled the chiropractor to see the impact of participating: *“I think that what the profession would want to see is that we want to see the real-world consequences of things.”* (P4—chiropractor).

### PBRN sub-study considerations

Within the sub-study model, it is important to complete a baseline study, providing member registration details, including clinician information, description of the clinic, and basic patient population information *“shows elements of practice and practice behaviours and practice shouldn't characteristics for the workforce*” (P10—researcher). At the end of the baseline study, participants are encouraged to consent to their contact details being stored on a database of practitioners for engagement and identification of practitioners for specific studies. However, another membership was concerning to one organisational representative, *“It's always a heart sink moment when I hear of yet another organization that's been that's been set up within chiropractic, you know.”* (P14—organisation).

Sub-study steering committees can be implemented to decide on approval of new research studies: *“we assessed all the sub studies that came through and made sure there were standard”* (P10—researcher). Prior PBRNs required studies to have both researchers and practitioners on the research team: “*it had to be led by an academic, but you had to had a have a co-investigator that was a clinician and the community that would help to facilitate and I think that worked really well. Just having a clinical lead who was, you know, kind of your advocate for the… PBRN.”* (P9—researcher).

Practitioners in the PBRN can decide on their involvement in each sub-study. Chiropractors reflected on wanting to participate in studies that were simple in design and how they decided to participate: *“How quick and easy it is to collect the information that is that for me that would be the sole thing that would sway it yes or no, is that, how easy it would be”* (P5—chiropractor). This was also noted by academics as an issue from previous research *“Researchers must be conscious of practitioner time, I think some of the barriers are gonna be time. They're gonna say how you know, how much time does this take? I've got too much admin as it is.”* (P12—academic).

Incentives were recommended to be a reasonable reimbursement for their time and effort, to increase participation and manage engagement with sub-studies: *“there has to be appropriate acknowledgement and reimbursement relative to the work that's being done, not to a point where it's overly incentivizing them”* (P10—researcher). Researchers highlighted this might be something small, *“it's just receiving something as a gift for recognition”* (P13—researcher). Examples from the qualitative interviews included a gift card acknowledgement and a raffle to win vouchers or wine. One researcher provided an example of providing recognition to involved clinics: *“we would send them some chocolates or flowers or whatever to say, hey, you're doing good, great. And maybe that's even more important than the payment that we really hear from many clinics”* (P20—researcher). However, organisations proposed financial reimbursement for larger studies: *“you'd need to be cognizant of the cost implications to the clinician and try to offset that would get better buy in”* (P27—organisation). In some cases, CPD points prove to be preferable and more enticing that monetary gifts, with chiropractors suggesting incentives such as *“Discount on the course or something like that, or you know, or a free online course that you can do”* (P5- chiropractor).

## Discussion

The aim of this project was to explore the feasibility and design of a PBRN for the chiropractic profession in the UK. The findings of this research have identified several benefits for the development of PBRNs. Real-world research can be enhanced through a PBRN. PBRNs can facilitate researchers and practitioners to produce relevant research to health care delivery [24]. A logic model (see Table 2) was developed to depict a PBRN within chiropractic, including the population, inputs and activities of the PBRN, and the outputs and outcomes. This research model enables collaboration between researchers and practitioners, helping to identify, establish and address research priorities focused on patient needs [39], providing pragmatic clinically relevant research from practice [6].

**Table 2:** Logic model of PBRNs

Population	Organisational input	Activities	Output	Outcomes
UK chiropractors—working in private practice (independent or within a group) UK chiropractic researchers (or those working within the field of musculoskeletal research) Chiropractic organisations (regulators, professional associations, professional membership bodies) Chiropractic patients	Advisory committee including director Administrator Chiropractors Practitioner advocates Researchers Sub-study steering committee Patient and public representatives	Recruitment of practitioners Baseline study Development of membership list Sub-study steering committees Meetings Regular communication (including research priority setting and dissemination)	Number of practitioners recruited Number of sub-studies Publications of sub-studies Number of meetings and network events Attendance at meetings at network events	Development of a membership list for future research Development of research active clinicians Increase in chiropractic research Dissemination of research and implementation of evidence into practice Improvements in patient care

The findings of this study identified several key elements to consider within the development of a UK-based PBRN for chiropractic. Our findings broadly reflect the models and structures reported in successful PBRNs internationally [17, 24–26]. and generally, tend to confirm rather than diverge from the wider literature on PBRN development and sustainability. The literature identified the two models of PBRNs as discussed in the interviews, the registry model and a sub-study model [25]. From the literature, chiropractors note a need for a patient outcome software integrated with clinical software alongside health records, currently used software required improvements to facilitate a registry model [36].The sub-study approach, as noted within the qualitative findings, is flexible and allows for a variety of study designs, including those utilising patient data records [25].

Specifically, the participants in this study emphasised the same core features consistently identified in established PBRNs. For example, a shared purpose [40, 41], robust governance and infrastructure beyond individual studies [13, 17, 20, 21, 23, 42], sustainable funding [16, 21, 43] and active strategies to support clinician engagement [19, 39, 44–47]. In. Our findings further suggest that in settings dominated by independent private practice such as UK chiropractic, these requirements may be even more important reinforcing the need for clear and explicit value propositions and central coordination to support long-term participation and network longevity. The findings of this work synthesise the learning from previous work in the chiropractic profession and bring insights from wider PBRN literature, to ensure sustainability of future PBRN development in the UK.

Despite noting the benefits of PBRNs and the positive impact on chiropractic research culture, participants identified multiple challenges. Issues identified included the model of PBRN delivery, with debates over the registry or sub-study model of PBRNs [25]. Although the registry model provides opportunities to collect routine patient data [24], the infrastructure required for this model is substantial and will require a significant change in the chiropractic profession. However, the sub-study model was identified as a relevant, pragmatic and useful model for a U.K. chiropractic PBRN. The model allows for the data to be explored by researchers and is a flexible approach for future studies, including those utilising patient data records [6, 25].

Questions were raised about the ownership of the PBRN an administration. There are three main types of organisational structure for a PBRN: operating via an academic institution, private organisations, and community-based participatory research [20]. In a 2011 review of 143 PBRNs registered with the Agency for Healthcare Research and Quality, 64.9% had a university as a primary affiliation, with 29% affiliated with a non-for-profit organisation [23]. Throughout the interviews, differing views were raised, with the consensus that an academic institution would have the infrastructure and ability to host a PBRN, however this should be run as an independent project with its own identity. Suggested infrastructure for PBRNs includes strategic leadership and administrative support, independent of a single study, to allow for the management and quality assurance of activity [48, 49]. Across PBRNs, strategic leadership often comes from a steering committee, to coordinate activity [21, 24, 50, 51]. Guidance on PBRN development suggests a research director takes overall responsibility for overseeing the network, including the management and operation of the PBRN through identifying practitioner advocates, writing marketing materials, recruitment of members, leading meetings, and launching projects [17, 19, 47], with administrative support [21, 43, 47].

PBRNs were noted to require sustainable funding [15], and it was recommended that PBRN development should not commence without this in place. In 2005, it was estimated the annual cost of a basic PBRN would be $69,700 (£55,000) with more complex networks costs estimated at $287,000 (£226,000) [43]. PBRNs are recommended to pursue alternative sources of funding to support infrastructure and activities. However, this must align with the mission and vision of the PBRN [16]. Funding could come from chiropractic associations, and this support has been seen from existing PBRNs. For example, the ACORN project was funded by the Chiropractors Association of Australia [24].

Previous research identified the issues in recruitment of chiropractors and their participation in research [35]. Noting that practitioners have limited time [19], recruitment of practitioners will continue to be a known issue with a PBRN. Significant investment needs to be made to involve practitioners at every level of the PBRN [17] and there must be ongoing engagement with the practitioners in the PBRN [15]. Previous recruitment activity has included emails to professional associations, news items for professional body newsletters, presentations at national and regional seminars and conferences, and a website providing an overview of the PBRN and the benefits and value to participating [17, 25]. Other recommendations included practitioner and public involvement in the sub-study steering committee and each sub-study team. Important potential benefits should be clearly communicated in the recruitment of chiropractors, including opportunities to contribute to research questions/projects, collect real-world data, work with researchers to implement new research findings in their practice, and be part of the network with access to other practitioners and research. Other recommendations from the literature included fun rewards such as food, gifts, or personalised thank you cards from the lead researcher [45]. Continuing professional development hours were another suggested incentive, which has been shown to be an incentive to participate [39]. Financial incentives have been shown to not increase participation but are associated with study completion [52]. In addition, a PBRN must manage the relationship with the practitioners in the network [20]. Recommendations for continued engagement include: an annual report, newsletters, presentations at network meetings, flyers, social media, videos, testimonials [19, 46, 47]. It is also recommended that ongoing engagement should facilitate PBRN members feedback [48], opportunity to develop research questions [53] and disseminate research findings [47].

One criticism of existing PBRNs is the lack of inclusion of patients within steering groups and leadership committees. A survey of 46 PBRNs in the U.S. across primary care networks found that 48% had no community involvement [54]. However, within Southern Primary-Care Urban Research Network (SPUR-NET), researchers meet with patients to incorporate patient perspectives into the research study design and processes [53]. It is recommended that individuals who are familiar with the chiropractic profession are recruited, to encourage patient participation within research, informing the research process from development to implementation [13]. Patient and participant involvement (PPI) should be an expectation of research undertaken in this new chiropractic PBRN.

### Strengths and limitations

There are several strengths and limitations to this study. Purposive sampling ensured a wide range of views were included in the study. Out of 33 researchers or representatives from organisations invited for interview, 25 took part in an interview. Only one organisation in the UK did not participate in an interview. Unfortunately, this highlights the difficulty of completing research and creating a PBRN in a fragmented profession. Due to social media recruitment, it is unknown how many chiropractors were invited to participate, and the small number of chiropractors who took part in the interview may represent only the views of chiropractors willing to take part in this type of research. This was supplemented by utilising existing literature on chiropractic involvement in research [35], which provides a comparison of participants to the wider chiropractic profession.

The interviews, coding and analysis were all conducted by two researchers, one a chiropractor and one with knowledge and engagement with the chiropractic profession. Although one researcher undertook the initial coding, this was later discussed with two researchers. Data interpretation and refinement of the constructs and theory was conducted in discussion across the team and considering the wider literature on PBRN development, to develop a comprehensive understanding of development of a PBRN in the chiropractic field.

### Recommendations

This research has identified core requirements for the development of PBRNs and provides a series of recommendations for PBRN development within the profession and within the development of a UK-based PBRN for chiropractic. This includes the use of a sub-study model, organisational structure requirements, securing sustainable funding, and advice on recruitment and engagement with chiropractors. These have been developed into one core model which can underpin the development and launch of PBRNs within the chiropractic profession. Within the UK, future work should focus on utilising the recommendations and resources for building a PBRN infrastructure to identify and build an advisory committee and secure sufficient funding. Further work should explore the inclusion of patients within PBRN and research committees, embedding the patient voice within the chiropractic research strategy.

## Conclusion

This study set out to explore and understand challenges in developing and maintaining a PBRN in the UK. There are several suggested benefits for a PBRN within chiropractic, including conducting real-world research, informing evidence-based practice and providing an infrastructure to develop the research culture. Challenges identified included the hosting of a PBRN, funding, and practitioner engagement. However, solutions and recommendations have identified approaches to recruitment, promotion and administration. The findings provide the chiropractic profession a clear strategy for development and maintenance of PBRNs.

## Supplementary Information

Below is the link to the electronic supplementary material.

Supplementary Material 1.

Supplementary Material 2.

## Data Availability

The datasets generated and/or analysed during the current study are not publicly available but are available from the corresponding author on reasonable request.
